# Three Days of Oral Azithromycin Versus Five Days of Oral Clarithromycin in the Treatment of Campylobacter Enterocolitis in Children: A Prospective Study

**DOI:** 10.3390/antibiotics13100969

**Published:** 2024-10-14

**Authors:** Hyun Mi Kang, Yoon Kyung Cho, Ye Ji Kim, Dae Chul Jeong, In Hyuk Yoo

**Affiliations:** 1 Department of Pediatrics, College of Medicine, The Catholic University of Korea, Seoul 06591, Republic of Korea; pedhmk@catholic.ac.kr (H.M.K.); yoonkcho1103@gamil.com (Y.K.C.); jenniferyejikim@gmail.com (Y.J.K.); dcjeong@catholic.ac.kr (D.C.J.); 2 Vaccine Bio Research Institute, College of Medicine, The Catholic University of Korea, Seoul 06591, Republic of Korea

**Keywords:** *Campylobacter* enterocolitis, azithromycin, clarithromycin, pediatric

## Abstract

Objective: This study aimed to compare the efficacy and tolerability of azithromycin and clarithromycin in pediatric *Campylobacter* enterocolitis. Methods: A prospective, randomized, controlled trial was conducted at a single center. Patients with confirmed *Campylobacter* enterocolitis were randomly assigned to receive either a 3-day course of azithromycin or a 5-day course of clarithromycin. Symptoms were monitored daily, and changes in laboratory markers (WBC counts, CRP levels, and stool calprotectin) were compared. Results: A total of 29 pediatric patients were included, with 14 patients in the azithromycin group and 15 patients in the clarithromycin group. The median age of patients in the azithromycin group was 10.0 years (interquartile range [IQR]: 5.0–13.0), and in the clarithromycin group, the median age was 9.0 years (IQR: 7.0–13.0) (*p* = 0.793). The median time to clinical resolution was 3.0 days (IQR: 2.0–3.0) in the azithromycin group and 2.0 days (IQR: 2.0–3.0) in the clarithromycin group (*p* = 0.132). There were no significant differences in the duration of individual symptoms, including fever, vomiting, and abdominal pain. The length of hospital stay was also similar, with a median stay of 4 days (IQR: 3.0–5.0) in both groups (*p* = 0.394). Both antibiotics were well-tolerated, with no significant adverse events or treatment discontinuation reported. Conclusions: Clarithromycin was found to be as effective as azithromycin in treating pediatric *Campylobacter* enterocolitis, with similar clinical outcomes and improvements in laboratory markers.

## 1. Introduction

*Campylobacter* enterocolitis is a significant cause of acute diarrheal illness worldwide, particularly affecting children and young adults. Among the species of *Campylobacter* (*C. jejuni*, *C. coli, C. upsaliensis, C. lari, C. concisus, C. fetus* subsp. *fetus, C. jejuni* subsp. *doylei,* and *C. hyointestinalis*) known to cause diarrhea, *Campylobacter jejuni* is the most prevalent, responsible for over 90% of reported cases, with *Campylobacter coli* accounting for most of the remaining infections [1]. *Campylobacter* enterocolitis is characterized by fever, diarrhea, and abdominal cramps, often accompanied by bloody stools [2]. These symptoms are not specific and are common across various bacterial causes of enterocolitis, making it challenging to distinguish *Campylobacter* enterocolitis from other bacterial infections based on clinical presentation alone [3].

In most cases, *Campylobacter* enterocolitis is a self-limited illness that resolves without the need for antibiotic therapy [2]. However, antibiotic treatment is indicated in pediatric patients with severe or complicated infections, such as those experiencing persistent fever, bloody diarrhea, more than eight bowel movements per day, prolonged symptoms lasting over seven days, suspected sepsis, disseminated infections, or those who are immunocompromised [1]. In these cases, antibiotic therapy has been shown to reduce the duration of symptoms and shorten the period of fecal excretion of *Campylobacter* bacteria [4,5]. Importantly, the efficacy of antibiotics is most pronounced when treatment is initiated within the first three days of symptom onset [5].

Historically, the delayed identification of *Campylobacter* as the causative agent often posed a barrier to the timely initiation of appropriate antibiotic therapy. Conventional stool culture methods or polymerase chain reaction (PCR) tests require several days to confirm the diagnosis, limiting the use of targeted antibiotics [6]. In clinical practice, this often resulted in empiric antibiotic therapy before results were confirmed, or in some cases, a delay in treatment entirely [7]. However, the development of rapid syndromic multiplex panels has revolutionized this process, enabling the identification of gastrointestinal pathogens, including *Campylobacter* species, within hours of stool collection [6,8]. This advancement has made the use of antibiotics more clinically relevant, particularly when therapy can be initiated within the critical three-day window after symptom onset [5].

*Campylobacter* species are inherently resistant to several classes of antibiotics, including trimethoprim and beta-lactams, such as penicillins and cephalosporins, making the choice of an effective antibiotic crucial [9]. Historically, erythromycin was considered the first-line treatment for *Campylobacter* enterocolitis, but concerns over drug interactions and side effects led to a shift in favor of azithromycin, particularly in pediatric patients. Most pediatric guidelines now recommend azithromycin as the standard treatment for *Campylobacter* enterocolitis, based on its efficacy and safety profile [3,10,11]. However, these recommendations are based on relatively limited evidence, primarily from studies in adult populations and a small number of pediatric studies [12,13,14].

Despite the widespread use of azithromycin, there has been little investigation into the efficacy of other macrolide antibiotics, such as clarithromycin, in treating pediatric *Campylobacter* enterocolitis. Clarithromycin is known to have similar antimicrobial properties to azithromycin and may offer advantages in certain clinical settings, such as availability and formulation options, particularly for pediatric patients. Given the limited data on its use in this population, there is a need for research to explore the potential role of clarithromycin in treating *Campylobacter* enterocolitis. The present study aimed to fill this gap by evaluating the efficacy and tolerability of a 5-day oral regimen of clarithromycin, compared with the standard 3-day course of azithromycin, in the treatment of pediatric *Campylobacter* enterocolitis.

## 2. Results

### 2.1. Patient Characteristics

**Figure 1:**
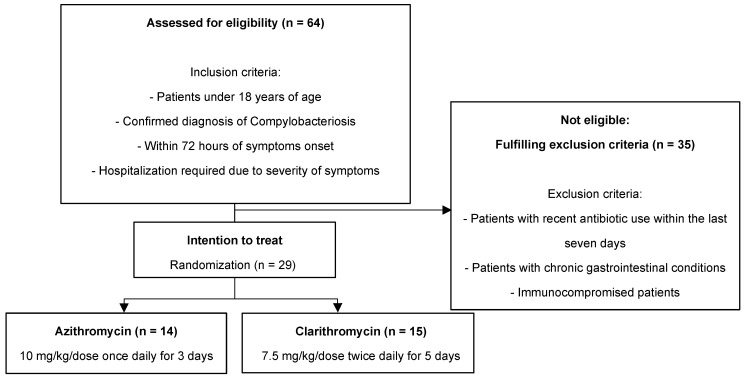
Study flow chart.

A total of 29 pediatric patients met the eligibility criteria and were randomly assigned to one of the two treatment groups, with 14 receiving azithromycin and 15 receiving clarithromycin (Figure 1). The baseline characteristics of the two groups are shown in Table 1, with no significant differences in terms of age, gender, or duration of illness prior to treatment. The median age was 10.0 (interquartile range [IQR]: 5.0–13.0) years in the azithromycin group and 9.0 (IQR: 7.0–13.0) years in the clarithromycin group (*p* = 0.793). The median duration of illness before initiating treatment was 2.5 (IQR: 2.0–4.0) days in the azithromycin group and 2.0 (IQR: 2.0–3.0) days in the clarithromycin group (*p* = 0.681). Additionally, laboratory parameters at the time of diagnosis, including WBC count, hemoglobin (Hb) levels, and CRP, were similar between the two groups, and the total symptom score was also comparable (Table 1).

### 2.2. Comparison of Clinical Outcomes

**Figure 2:**
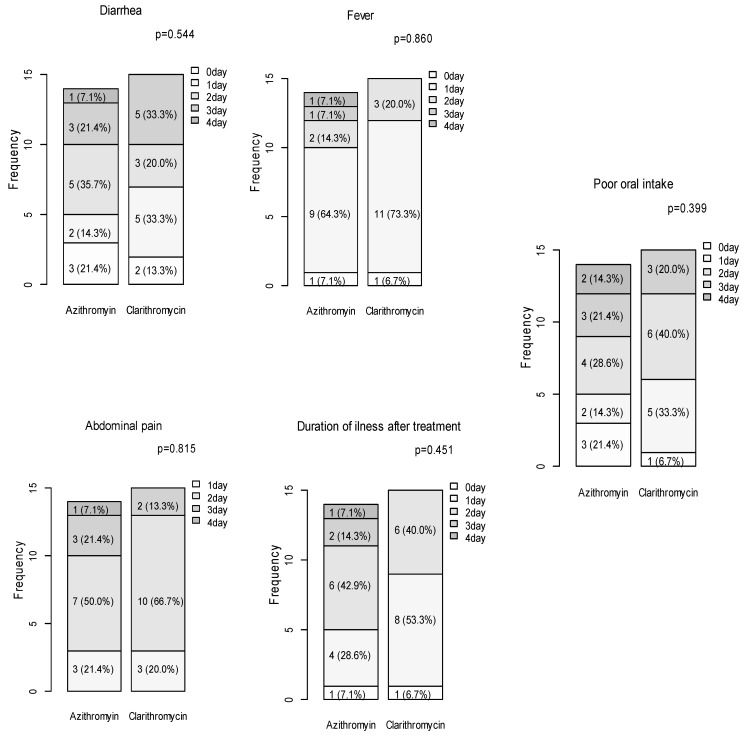
Comparison of days to recovery for individual clinical symptoms and duration of illness in the azithromycin versus clarithromycin groups.

The primary outcome, time to clinical resolution, was also comparable between the two treatment arms. The median time to clinical resolution was 3.0 (IQR: 2.0–3.0) days in the azithromycin group and 2.0 (IQR: 2.0–3.0) days in the clarithromycin group, with no statistically significant difference (*p* = 0.132). This similarity was observed across individual symptoms as well. The duration of fever (*p* = 0.575), vomiting (*p* = 0.597), abdominal pain (*p* = 0.555), and poor oral intake (*p* = 0.668) did not significantly differ between the groups. For instance, the median duration of fever was 1 day for both groups (*p* = 0.575), while the median duration of abdominal pain was 2 days for both groups (*p* = 0.555). Similarly, no difference was observed in the duration of diarrhea (*p* = 0.946). The length of hospital stay was also comparable between the two groups. The median stay was 4 days for both the azithromycin and clarithromycin groups (*p* = 0.394) (Table 2). Furthermore, when comparing the two groups based on the number of days required for recovery from each clinical symptom and overall clinical status, no significant differences were observed between the groups (Figure 2).

### 2.3. Comparison of Laboratory Outcomes

In terms of laboratory outcomes, there was a significant reduction in the WBC count from day 1 to day 3 in both treatment groups. The median WBC count in the azithromycin group decreased from 7985.0 (IQR: 7300.0–12,910.0) cells/µL at baseline to 6165 (IQR: 5016.0–6730.0) cells/µL by day 3 (*p* < 0.001). Similarly, in the clarithromycin group, the WBC count dropped from 10,260.0 (IQR: 7110.0–14,100.0) cells/µL at baseline to 5730 (IQR: 4100.0–7390.0) cells/µL by day 3 (*p* < 0.001) (Table 3). However, the magnitude of this reduction did not differ significantly between the groups (*p* = 0.647) (Table 4). Likewise, CRP levels showed a marked decrease in both groups by day 3, with a slightly greater reduction in the clarithromycin group. However, this difference was not statistically significant (*p* = 0.275) (Table 3 and Table 4).

Regarding stool calprotectin levels, which serve as a marker of intestinal inflammation, both groups exhibited significant reductions between day 1 and day 3. The median calprotectin level in the azithromycin group dropped from 470.5 µg/g to 146.0 µg/g (*p* = 0.006), while in the clarithromycin group, the level decreased from 833.5 µg/g to 205.5 µg/g (*p* = 0.004). While the clarithromycin group showed a slightly larger reduction, the difference between the groups was not statistically significant (*p* = 0.748).

### 2.4. Drug Tolerability

No significant adverse events were reported in either group, with both antibiotics being well tolerated by the patients without any discontinuation of treatment.

## 3. Discussion

This study aimed to compare the clinical efficacy and tolerability of azithromycin and clarithromycin in pediatric *Campylobacter* enterocolitis, with the findings indicating that both antibiotics are similarly effective. Our results demonstrate that there were no significant differences between the two groups in terms of time to clinical resolution, symptom duration, or length of hospital stay, confirming the comparable efficacy of both treatments. The median time to clinical resolution was 3.0 days for azithromycin and 2.0 days for clarithromycin, a difference that was not statistically significant (*p* = 0.132). Additionally, both treatment groups showed significant improvements in WBC counts, CRP levels, and stool calprotectin, though no statistically significant differences were observed between the groups for these laboratory parameters. Importantly, both antibiotics were well-tolerated, with no adverse events requiring treatment discontinuation.

Previous studies, including a meta-analysis, have established the importance of early antibiotic intervention in pediatric *Campylobacter* enterocolitis, particularly when initiated within the first three days of symptom onset, as this approach has been shown to significantly shorten the duration of illness and reduce fecal excretion of *Campylobacter* [2,5]. Consistent with this evidence, both azithromycin and clarithromycin demonstrated comparable effectiveness in reducing the median time to clinical resolution in our study. The median time to resolution was 3.0 days for azithromycin and 2.0 days for clarithromycin, a difference that was not statistically significant (*p* = 0.132).

Azithromycin is currently recommended as the first-line treatment for *Campylobacter* enterocolitis, particularly in pediatric populations, based on its efficacy and safety profile [3,11]. However, comparative studies investigating the use of other macrolides, such as clarithromycin, remain limited [12,13,14]. While azithromycin remains the preferred treatment in most pediatric guidelines, clarithromycin has shown potential as an alternative. The European Committee on Antimicrobial Susceptibility Testing (EUCAST) has indicated that *Campylobacter jejuni* and *Campylobacter coli* strains susceptible to erythromycin are generally also susceptible to azithromycin and clarithromycin [15,16]. Despite this, clarithromycin is not widely recommended due to the lack of robust clinical studies assessing its use in pediatric *Campylobacter* enterocolitis. Our study provides initial evidence that clarithromycin may offer a viable alternative to azithromycin, particularly in cases where azithromycin is unavailable or unsuitable.

Beyond clinical symptoms, our study also examined key laboratory outcomes, including WBC counts, CRP levels, and stool calprotectin levels, to assess the impact of both antibiotics on systemic and intestinal inflammation. Both groups exhibited significant reductions in these markers by day 3, further supporting the clinical effectiveness of both antibiotics. WBC counts and CRP levels showed marked reductions in both groups, with no statistically significant differences in the magnitude of these changes. Similarly, stool calprotectin, a reliable marker of intestinal inflammation, decreased significantly in both groups, but the difference in the reduction was not statistically significant (*p* = 0.748). This finding further supports the hypothesis that clarithromycin and azithromycin offer similar benefits when started early in the course of pediatric *Campylobacter* enterocolitis.

Both antibiotics were well-tolerated, with no significant adverse events reported. This is particularly important in pediatric populations, where the safety profile of antibiotics is a critical consideration. Our findings align with previous research indicating that macrolide antibiotics are generally well-tolerated in children [9]. Given the increasing importance of antibiotic stewardship [17,18,19] and the need to provide multiple treatment options in pediatric infectious diseases, the availability of clarithromycin as a treatment option could be valuable in clinical practice, especially in cases where azithromycin may be contraindicated or unavailable. Additionally, a study found no significant differences between azithromycin and clarithromycin regarding their impact on human oropharyngeal and intestinal microflora. Neither antibiotic was linked to colonization by resistant Gram-positive bacteria or the overgrowth of opportunistic microorganisms [20,21,22,23]. This suggests that, when used appropriately, both antibiotics have minimal impact on the microbiota.

Although our study provides valuable insights into the comparative efficacy of azithromycin and clarithromycin, it is not without limitations. This study was conducted at a single center with a relatively small sample size, limiting the generalizability of our findings. The sample size was also insufficient to demonstrate the non-inferiority of clarithromycin with statistical certainty. Future studies with larger sample sizes and multi-center trials are necessary to confirm our findings and explore the potential role of clarithromycin in pediatric *Campylobacter* enterocolitis. Another limitation was that the laboratory values obtained on the third day may be too early to accurately reflect the full benefits of the antibiotics. Additionally, further research could explore the cost-effectiveness of these treatment options, particularly in resource-limited settings where access to specific antibiotics may be constrained.

## 4. Materials and Methods

### 4.1. Study Design

This study was designed as a prospective, randomized, controlled trial aimed at comparing the clinical outcomes and changes in laboratory parameters between two treatment groups—azithromycin and clarithromycin—for *Campylobacter* enterocolitis in pediatric patients. This study was conducted in a single center and adhered to standard ethical guidelines, including obtaining informed consent from the guardians of all participating patients. Patients who were eligible for this study were randomly assigned to receive one of the two treatment regimens using a computer-generated randomization process. The study design was approved by the institutional ethics committee of Seoul St. Mary’s Hospital (IRB no. KC22TISI0390) and followed the principles outlined in the Declaration of Helsinki. Signed consent forms were obtained from all patients and/or their legal guardians.

### 4.2. Inclusion and Exclusion Criteria

Patients below 18 years of age diagnosed with a clinical and laboratory-confirmed diagnosis of Campylobacteriosis, only probable and confirmed cases that presented within 72 h of symptom onset, and those who required hospitalization due to the severity of their symptoms, such as multiple episodes of diarrhea or dehydration, were included. In this study, a case of campylobacteriosis was defined when a patient presented with symptoms suggestive of acute enterocolitis, such as diarrhea, abdominal pain, hematochezia, or vomiting, and had laboratory confirmation of Campylobacter in their initial stool samples. Probable cases were defined as cases with symptoms plus *Campylobacter* species detected using culture-independent diagnostic tests (e.g., PCR), and confirmed cases were defined in patients with symptoms plus *Campylobacter* cultured from stools [24].

Patients with a history of recent antibiotic use within the last seven days, chronic gastrointestinal conditions, or immunocompromised status were excluded from this study to ensure that any treatment effects observed could be attributed to the intervention. The intervention for the azithromycin group involved administering a 3-day course of oral azithromycin. The dosage was 10 mg/kg/dose once daily. In contrast, the clarithromycin group received a 5-day course of oral clarithromycin, with a dosage of 7.5 mg/kg administered twice daily. Both medications were initiated within 72 h of the onset of symptoms. All patients were monitored daily for the following: vomiting, abdominal pain, nausea, diarrhea frequency, blood in stool, watery stool, fever, poor food intake (none or mild: two-thirds to the full usual amount; moderate: one-third to two-thirds of the usual amount; and severe: less than one-third of the usual amount), and total symptom score. The total symptom score was calculated by combining the number of daily stools, presence of dysenteric stools, presence of watery stools, maximal daily temperature, presence of vomiting, and dehydration. This score was used to assess the ‘baseline disease severity’. The maximum clinical score was 10, and the minimum score was 0, as detailed in Leibovitz et al.’s study [25]. Laboratory tests were performed at the initial visit (day 1) and on day 3 of admission.

### 4.3. Outcome Measures

The primary outcome of this study was to compare the two intervention groups (azithromycin vs. clarithromycin groups) in terms of the time to clinical resolution, defined as the complete disappearance of fever, diarrhea, abdominal pain, and a return to normal oral intake. The secondary outcomes included the duration of each of these symptoms individually, the overall length of hospital stay, and changes in laboratory markers, including white blood cell (WBC) counts, C-reactive protein (CRP) levels, and stool calprotectin levels, all of which were measured at baseline and again on the third day of treatment. The proportion of patients with positive stool calprotectin results was also monitored.

### 4.4. Statistical Analyses

For paired data, the Wilcoxon signed-rank test was used to assess differences in continuous variables, while McNemar’s test was used for categorical variables to evaluate changes between paired observations. For unpaired data, the Wilcoxon rank-sum test was applied to compare non-normally distributed continuous variables between the two treatment groups. Categorical data were analyzed using the chi-square test or Fisher’s exact test, depending on the expected frequencies in contingency tables. A *p*-value of <0.05 was considered indicative of statistical significance in all tests. All statistical analyses were performed using the SPSS software (version 24.0; IBM Corp., Armonk, NY, USA).

## 5. Conclusions

In conclusion, this study demonstrated that clarithromycin is as effective as azithromycin in treating pediatric *Campylobacter* enterocolitis, with no significant differences in clinical outcomes, laboratory markers, or drug tolerability. These findings suggest that clarithromycin could serve as a viable alternative to azithromycin, broadening the range of treatment options available for this common pediatric infection. However, larger multi-center studies are needed to confirm the equivalence of these two macrolide antibiotics and provide further evidence to support the incorporation of clarithromycin into treatment guidelines for *Campylobacter* enterocolitis.

## Figures and Tables

**Table 1:** Baseline characteristics.

Variable	Azithromycin (*n* = 14)	Clarithromycin (*n* = 15)	*p*
Male, no (%)	9 (64.3)	8 (53.3)	0.550
Female, no (%)	5 (35.7)	7 (46.7)	
Age, years	10.0 (5.0–13.0)	9.0 (7.0–13.0)	0.793
Duration of illness before treatment, days	2.5 (2.0–4.0)	2.0 (2.0–3.0)	0.681
Laboratory parameters at diagnoses			
WBC count	7985.0 (7300.0–12,910.0)	10,260.0 (7110.0–14,100.0)	0.647
Hb	13.1 (12.0–13.8)	13.0 (12.6–14.3)	0.347
CRP	7.2 (2.7–10.8)	8.2 (4.4–10.2)	0.556
BUN	10.8 (7.6–12.0)	9.1 (8.1–10.5)	0.156
Cr	0.5 (0.4–0.6)	0.5 (0.4–0.6)	0.965
AST	21.0 (17.0–30.0)	27.0 (18.0–30.0)	0.497
ALT	15.5 (12.0–23.0)	15.0 (10.0–21.0)	0.727
Total bilirubin	0.4 (0.3–0.5)	0.3 (0.3–0.4)	0.948
Na	136.5 (135.0–138.0)	135.0 (132.0–137.0)	0.222
K	4.2 (3.9–4.4)	4.1 (4.1–4.3)	0.808
Cl	102.0 (101.0–104.0)	100.0 (99.0–102.0)	0.095
Stool occult blood positive, no (%)	8 (57.1)	8 (53.3)	0.837
Stool pus cell positive, no (%)	4 (28.6)	5 (33.3)	>0.999
Stool calprotectin level	470.5 (119.0–1000.0)	833.5 (408.0–950.0)	0.908
Stool culture positive, no (%)	6 (42.9)	10 (66.7)	0.198
Distribution of patients by symptoms at diagnoses			
Vomiting, no (%)	6 (42.9)	5 (33.3)	0.597
Abdominal pain, no (%)	14 (100.0)	14 (93.3)	>0.999
Nausea, no (%)	6 (42.9)	7 (46.7)	0.837
Diarrhea (frequency per day)			0.695
None, no (%)	0 (0.0)	2 (13.3)	
1–3, no (%)	3 (21.4)	2 (13.3)	
4–6, no (%)	6 (42.9)	5 (33.3)	
>7, no (%)	5 (35.7)	6 (40.0)	
Blood in stool, no (%)	3 (21.4)	4 (26.7)	>0.999
Watery stool, no (%)	14 (100.0)	13 (86.7)	0.483
Fever, no (%)	13 (92.9)	15 (100.0)	0.483
Poor food intake			0.782
None, no (%)	1 (7.1)	1 (6.7)	
Mild, no (%)	3 (21.4)	1 (6.7)	
Moderate, no (%)	4 (28.6)	6 (40.0)	
Severe, no (%)	6 (42.9)	7 (46.7)	
Total symptom score	5.0 (4.0–6.0)	5.0 (3.0–6.0)	0.965

Non-parametric numbers are shown as medians (interquartile ranges). *p*-values were calculated by the chi-square test, Fisher’s exact test, or the Wilcoxon rank-sum test.

**Table 2:** Duration of abnormal symptoms after treatment associated with Campylobacteriosis.

	Azithromycin (*n* = 14)	Clarithromycin (*n* = 15)	*p*
Fever	1.0 (1.0–2.0)	1.0 (1.0–1.0)	0.575
Vomiting	6 (42.9)	5 (33.3)	0.597
Abdominal pain	2.0 (2.0–3.0)	2.0 (2.0–2.0)	0.555
Diarrhea	2.0 (1.0–3.0)	2.0 (1.0–3.0)	0.946
Poor oral intake	2.0 (1.0–3.0)	2.0 (1.0–2.0)	0.668
Duration of illness *	3.0 (2.0–3.0)	2.0 (2.0–3.0)	0.132
Admission duration	4.0 (4.0–4.0)	4.0 (3.0–4.0)	0.394

All values are shown as median days (interquartile ranges). *p*-values were calculated by the chi-square test, Fisher’s exact test, or the Wilcoxon rank-sum test. * The duration of illness refers to the total period encompassing fever, vomiting, abdominal pain, diarrhea, and poor oral intake.

**Table 3:** Changes in laboratory parameters after treatment.

	Azithromycin (*n* = 14)			Clarithromycin (*n* = 15)		
	Day 1	Day 3	*p*	Day 1	Day 3	*p*
WBC count, cells/µL	7985.0 (7300.0–12,910.0)	6165.0 (5016.0–6730.0)	<0.001	10,260.0 (7110.0–14,100.0)	5730.0 (4100.0–7390.0)	<0.001
Hb, g/dL	13.1 (12.0–13.8)	12.9 (12.0–13.7)	0.660	13.0 (12.6–14.3)	12.3 (11.6–14.1)	0.005
CRP, g/dL	7.2 (2.7–10.8)	1.4 (0.3–6.1)	0.008	8.2 (4.4–10.2)	1.4 (0.9–5.1)	<0.001
BUN, g/dL	10.8 (7.6–12.0)	7.1 (5.5–8.3)	0.003	9.1 (8.1–10.5)	5.8 (3.5–8.0)	0.002
Cr, g/dL	0.5 (0.4–0.6)	0.5 (0.4–0.5)	<0.001	0.5 (0.4–0.6)	0.4 (0.4–0.5)	0.003
AST, g/dL	21.0 (17.0–30.0)	18.0 (16.0–28.0)	0.990	27.0 (18.0–30.0)	20.0 (18.0–28.0)	0.164
ALT, g/dL	15.5 (12.0–23.0)	12.0 (11.0–28.0)	0.912	15.0 (10.0–21.0)	15.0 (11.0–19.0)	0.605
Total bilirubin, g/dL	0.4 (0.3–0.5)	0.2 (0.2–0.3)	<0.001	0.3 (0.3–0.4)	0.3 (0.2–0.3)	0.003
Na, g/dL	136.5 (135.0–138.0)	140.0 (139.0–141.0)	0.003	135.0 (132.0–137.0)	139.0 (138.0–140.0)	<0.001
K, g/dL	4.2 (3.9–4.4)	4.2 (3.8–4.3)	0.693	4.1 (4.1–4.3)	3.9 (3.8–4.1)	0.222
Cl, g/dL	102.0 (101.0–104.0)	105.5 (105.0–107.0)	0.017	100.0 (99.0–102.0)	106.0 (105.0–107.0)	<0.001
Stool occult blood, no (%)	8 (57.1)	2 (14.3)	0.014	8 (57.1)	1 (7.1)	0.008
Stool pus cell, no (%)	4 (28.6)	1 (7.1)	0.083	5 (35.7)	2 (14.3)	0.083
Stool calprotectin level	470.5 (119.0–1000.0)	146.0 (51.0–688.0)	0.006	833.5 (408.0–950.0)	205.5 (120.0–511.0)	0.004
Proportion of patients positive for stool calprotectin, no (%)	10 (71.4)	6 (42.9)	0.046	11 (78.6)	7 (50.0)	0.046

*p*-values were calculated using the Wilcoxon signed rank-sum test or McNemar’s test for paired data, and the Wilcoxon rank-sum test, the chi-square test, or Fisher’s exact test for unpaired data.

**Table 4:** Comparison of changes in laboratory parameters between the azithromycin and clarithromycin groups.

ΔDay 3–Day 1	Azithromycin (*n* = 14)	Clarithromycin (*n* = 15)	*p*-Value
ΔWBC count, cells/µL	−3172.0 (−5080.0, −1600.0)	−4010.0 (−6200.0, −1560.0)	0.647
ΔHb, g/dL	0.0 (−0.3, 0.2)	−0.4 (−1.3, −0.1)	0.088
ΔCRP, g/dL	−1.7 (−9.3, −0.3)	−5.8 (−8.8, −2.2)	0.275
ΔBUN, g/dL	−2.9 (−6.6, −0.4)	−3.2 (−5.5, −0.5)	>0.999
ΔCr, g/dL	−0.1 (−0.1, −0.1)	−0.1 (−0.2, 0.0)	0.983
ΔAST, g/dL	−1.0 (−6.0, 5.0)	−1.0 (−9.0, 1.0)	0.555
ΔALT, g/dL	−0.5 (−4.0, 4.0)	0.0 (−3.0, 1.0)	0.742
ΔTotal bilirubin, g/dL	−0.1 (−0.2, −0.1)	−0.1 (−0.2, −0.1)	0.445
ΔNa, g/dL	4.5 (1.0, 6.0)	4.0 (1.0, 7.0)	0.758
ΔK, g/dL	0.0 (−0.4, 0.1)	−0.3 (−0.5, 0.2)	0.57
ΔCl, g/dL	4.0 (1.0, 5.0)	4.0 (3.0, 8.0)	0.253
ΔStool calprotectin levels, µg/g	−167.5 (−467.0, −40.0)	−325.5 (−606.0, −33.0)	0.748

Values are shown as numbers. *p*-values were calculated by the Wilcoxon rank-sum test.

## Data Availability

The data presented in this study are available upon request from the corresponding author due to privacy concerns.
